# The Essential Functions of NEDD8 Are Mediated via Distinct Surface Regions, and Not by Polyneddylation in *Schizosaccharomyces pombe*

**DOI:** 10.1371/journal.pone.0020089

**Published:** 2011-05-31

**Authors:** David Girdwood, Dimitris P. Xirodimas, Colin Gordon

**Affiliations:** 1 Medical Research Council Human Genetics Unit, Western General Hospital, Edinburgh, Scotland, United Kingdom; 2 Wellcome Trust Centre for Gene Regulation and Expression, College of Life Sciences, University of Dundee, Dundee, Scotland, United Kingdom; Karolinska Institutet, Sweden

## Abstract

The ubiquitin-like protein NEDD8 is highly conserved in eukaryotes, from man to *Schizosaccharomyces pombe*. NEDD8 conjugation to cullin proteins is a prerequisite for cullin based E3 ubiquitin ligase activity, and essential for *S. pombe* viability. Here, we have performed alanine scanning mutagenesis of all conserved surface residues and show that the majority of essential residues were located around the hydrophobic patch and the C-terminus. However, we further identified essential residues not previously reported to be involved in ubiquitin ligase regulation that importantly do not prevent Ned8p conjugation. We also find that mutation of all conserved lysine residues in Ned8p, did not affect yeast viability, suggesting that mono-neddylation is sufficient for yeast viability under most conditions.

## Introduction

Post-translational modification by the covalent attachment of NEDD8 to acceptor lysines, is an essential biological process in the majority of eukaryotic organisms, with the notable exception of *Saccharomyces cerevisiae*. This process of NEDD8 conjugation, termed neddylation, has been reported for a diverse range of substrates [1]. Neddylation is achieved in a manner analogous to that of ubiquitination, in which distinct E1 and E2-like enzymes activate NEDD8 for subsequent conjugation via a limited number of E3-like enzymes. A sole E3-like enzyme specific to the NEDD8 conjugation pathway, DCN1, has been reported [2]. In addition, the ubiquitin ligases Mdm2 and Roc1 have been reported to have dual specificity and can also function as NEDD8 ligases, as they were shown to neddylate p53 and Cullin1 respectively [3], [4]. Removal of conjugated NEDD8 from substrates is achieved via the actions of the cysteine protease NEDP1/DEN1 [5], [6] and the metalloprotease activity of the CSN5 subunit of the COP9/signalosome complex [7].

To date, the most documented role for NEDD8 is through its modification of the cullin family of proteins. Neddylation of a cullin protein stimulates the activity of the cullin-RING E3 ligase (CRL) *in vitro* [8] and is essential for activity *in vivo* [9]. This positive influence on CRL activity is achieved by NEDD8 acting in an antagonistic manner to the binding of a complex inhibitor, CAND1, to the CRL complex [10], NEDD8 modification is further involved in regulating CRL activity by enhancing the recruitment of ubiquitin-activated E2s [11], and by inducing a major conformational change in the CRL complex, such that a 50 Å gap between the substrate and the ubiquitin-loaded E2 is reduced [12], [13].

NEDD8 has the highest percentage identity to ubiquitin, out of all the ubiquitin-like family of proteins. The conservation of these residues includes many that are known to be essential for ubiquitin function, such as Lys48 and Ile44. In ubiquitin, Lys48, is essential for the formation of ubiquitin chains, which are subsequently recognised by the 26S proteasome [14], resulting in the degradation of the modified substrate. The recognition of ubiquitin is mediated via the canonical “hydrophobic patch”, which consists of Leu8, Ile44, and Val70. Interactions with ubiquitin are not restricted to the hydrophobic patch, as important residues for ubiquitin-protein interaction are dispersed over its globular surface [15], [16], [17], [18].

The ability of ubiquitin to conjugate via internal lysine residues other than Lys48 greatly enhances the functional consequences of ubiquitination. Ubiquitin chains branching from Lys63 have been implicated in numerous biological processes, and budding yeast lacking the ability to form Lys63 chains have DNA damage sensitivities [19], [20], [21], [22], [23]. The versatility of ubiquitin as a regulator of intracellular pathways is therefore mediated by the presentation of distinct surface topologies on ubiquitin chains which allows for distinct functional consequences following attachment, [24], [25]. NEDD8 has also been reported to form chains *in vivo* [26], [27], although the function of polyneddylation remains to be determined. NEDD8, like ubiquitin, contains the evolutionary conserved canonical hydrophobic patch, and the ability of NEDD8 to interact and recruit UBC4 is mediated by this protein binding site [11]. In order to identify the important regions on the surface of Ned8p, we performed alanine scanning mutagenesis of the *S. pombe ned8^+^*. We mutated all evolutionally conserved residues at the surface of Ned8p, to map the residues that could function in mediating protein-protein interactions. We report that polyneddylation is not essential for viability, and identify residues essential for Ned8p function, but not conjugation.

## Results

### Ned8p residues essential for viability

**Figure 1:**
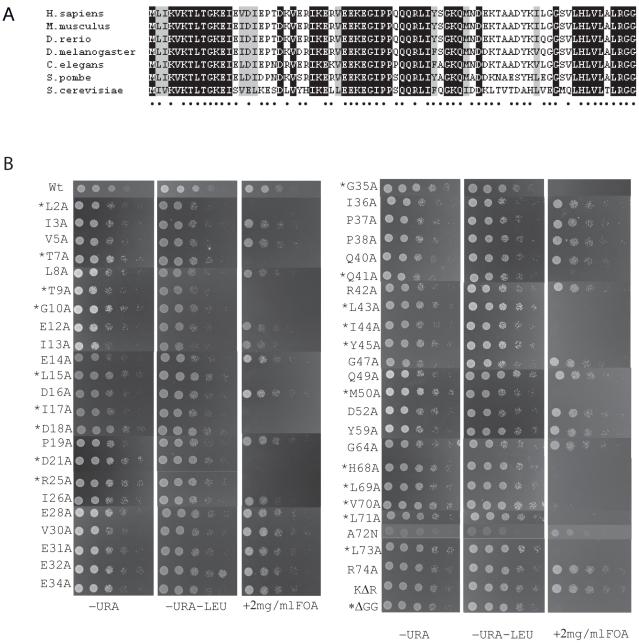
Essential Ned8 residues. A. Sequence alignments of *H. sapiens*, *M. musculus*, *D. rerio*, *D. melanogaster*, *C. elegans*, *S. pombe*, *S. cerevisiae*. Surface residues are marked by closed circles (•). Black boxes indicate identical residues and grey boxes, conservative substitutions. B. Growth assay of serially diluted strains bearing an integrated alanine mutation of Ned8 (as indicated) and assessed for growth on PMG-leucine (−LEU), PMG-leucine-uracil (PMG-LEU-URA), or PMG medium lacking leucine but supplemented with 5-FOA (−LEU +FOA), which is toxic to *ura4^+^* cells. * indicates essential residues.

**Figure 2:**
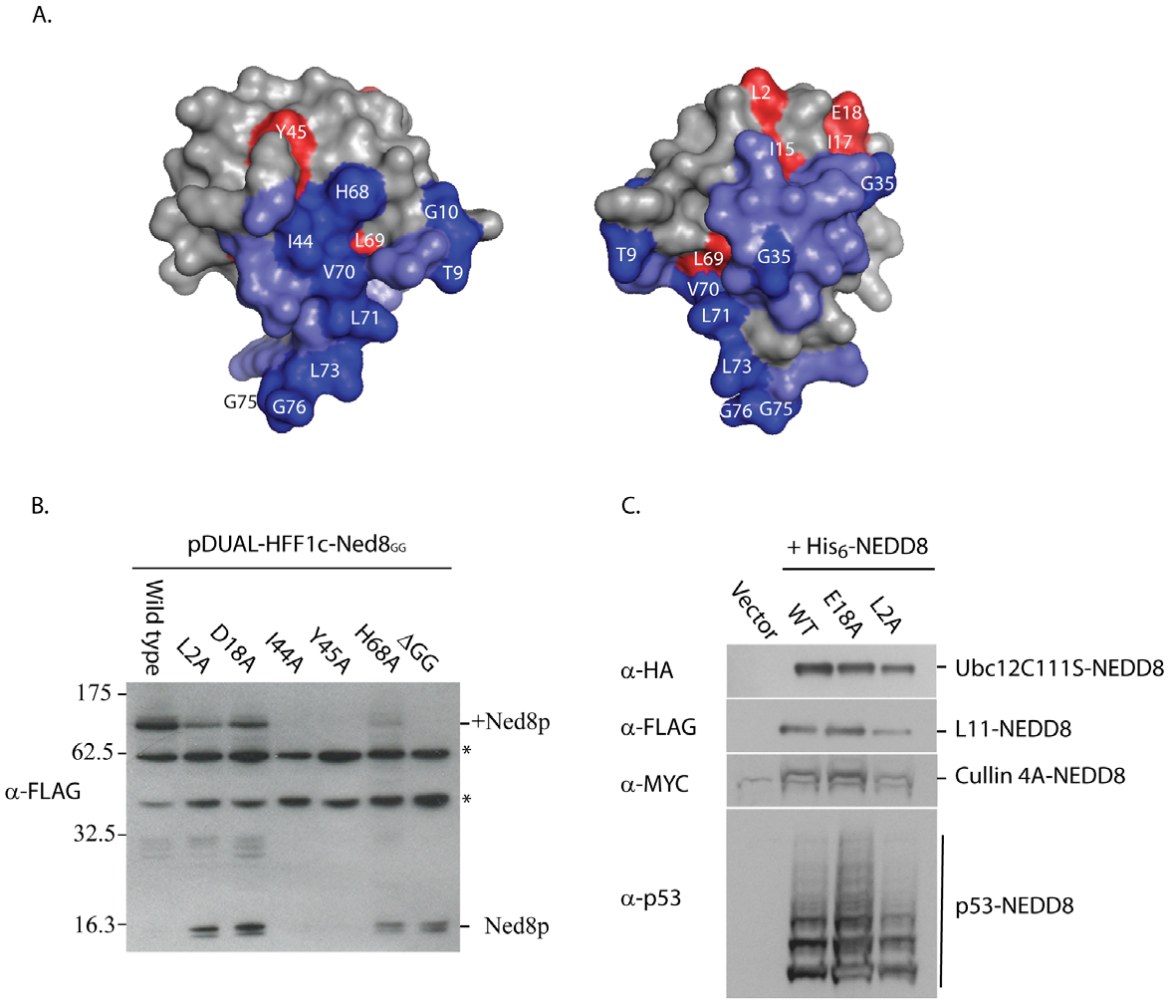
Conjugation of mutant Nedp constructs. A. Space-filling representation of residues on NEDD8 known to be involved in protein-protein interactions. Residues involved in reported protein-protein interactions are indicated in light blue for non-essential and dark blue for essential. Essential residues not known to be involved in interactions are coloured red. Figures were made using MacPyMOL on NEDD8 (PDB 1NDD). B. S. pombe strains expressing pDUAL-HFF1c-Ned8GG, and indicated mutants. Total cell lysates (10^8^ cells) were prepared under denaturing conditions, and 6 µl of each extract were separated on a 10% SDS-PAGE gel, prior to the blot being probed with anti-FLAG monoclonal antibody. *denotes non-specific band. C. H1299 cells were transfected His_6_-NEDD8 wild-type and mutants along with HA-Ubc12C111S, Flag-L11, p53 and Myc-Cullin 4 as indicated.

NEDD8 is highly conserved in eukaryotes, with ∼80% identity between that of the *H. sapiens* and *S. pombe* orthologues. To identify surface and solvent accessible residues (as identified from the three dimensional structure [28]) required for viability, we performed comprehensive alanine scaning mutagenesis of these conserved and semi-conserved residues (Fig. 1a). Plasmids encoding mutant constructs of *ned8* were integrated into a strain carrying an episomal plasmid (*ura4*) containing wild type *ned8^+^* and deleted for endogenous *ned8^+^*. Selection against the version by 5-FOA allowed us to identify which of the Ned8p mutants are sufficient for viability, as assayed for growth at 25°C (Fig. 1b). Viable Ned8p mutants were then further subjected to temperature stress at 36°C and cold sensitivity at 20°C. Of the forty-one evolutionary conserved and semi-conserved surface residues fourteen were essential, and none displayed conditional growth phenotypes. To date, the only known protein-protein interactions with NEDD8 are through its interactions with its cognate conjugation and deconjugation enzymes [29], [30], [31], and the ubiquitin E2, UBC4 [11]. These known sites of contacts were mapped onto a three-dimensional structure of NEDD8 [28]: and indicated in blue. Following discrimination of the residues found to be non-essential (light blue) and essential (dark blue) for these contacts are indicated (Fig. 2a). Novel essential residues, not known to be required for conjugation are indicated in red (Fig. 2a). To address whether the loss of viability was due to the inability of the mutant Ned8p to conjugate, selected mutants were remade in N-terminally tagged vectors and transformed into wild type *S. pombe*. The strains carrying the L2A, D18A, H68A mutations were all capable of forming, albeit somewhat reduced, higher molecular weight adducts, although no conjugation was detectable for Ile44 or Tyr45 mutants (Fig. 2b). This lack of conjugation could be explained by the stability of the proteins *in vivo* as no unconjugated Ned8p of the mutants were detected.

To further investigate this observation, regarding the capacity of the Ned8p mutants to conjugate, we utilised a mammalian system. Transfection of plasmids containing His_6_-Human NEDD8 (wild type, L2S, or E18A) into H1299 cells, showed conjugation to a number of previously characterised substrates (Fig. 2c) [3], [26], [32], [33]. These results show that the L2A and D18A mutations do not affect the ability of NEDD8 to conjugate, but instead affect another aspect of Ned8p function that is essential for viability.

### Polyneddylation is not essential for *S. pombe* viability

Strikingly, the Ned8p mutant, in which all lysine residues had been mutated to arginine (K0), was also viable (Fig. 1b). Although Western blot analysis of the tagged Ned8p, did not reveal any “ladder” of Ned8p conjugation, this is likely explained by the prominence of cullin(s) modification being readily detected by Western blot analysis, as mass spectrometrical analysis indicated the *in vivo* occurrence on at least some internal lysine residues (data not shown). Additionally we tested whether polyneddylation might be involved in the stress response. Growth of the two strains was compared in the presence of a range of poisons, including DNA damaging agents (methyl methanesulfonate, camptothecin, and hydroxyurea), a microtubule poison (thiabendazole), and proteasome stress inducing reagents (cadmium chloride and l-canavanine). However, we detected no significant differences in growth between the two strains for any of the drugs tested (data not shown).

### Comparison of the Ned8p and Ubiquitin residues essential for viability

**Figure 3:**
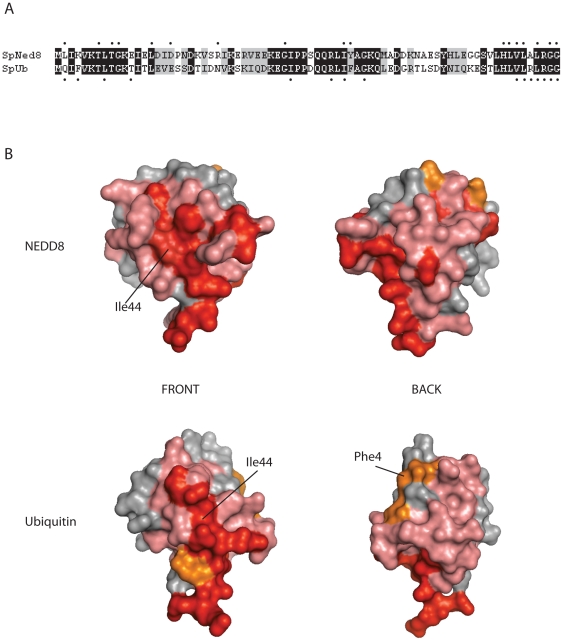
Essential surface residues of NEDD8 and ubiquitin. A. Alignment of sequences of *S. pombe* Nedp and ubiquitin. Closed circles (•) above Nedp and below ubiquitin denote the essential surface residues. Black boxes indicate identical residues and grey boxes, conservative substitutions. B. Space-filling representation of essential surface residues on NEDD8 and ubiquitin. Non-essential conserved residues are shown in light red, essential conserved in red, and essential non-conserved residues in orange, and two views of NEDD8 and ubiquitin are shown. Figures were made using MacPyMOL on NEDD8 (PDB 1NDD) and ubiquitin (PDB 1UBQ).

As expected from the high sequence and structural similarities between Ned8p and ubiquitin there was significant overlap in the essential residues identified (Fig. 3a) [18]. Conserved, essential (red) and non-essential residues (light red) were mapped onto three dimensional structures of both NEDD8 and ubiquitin [34] (Fig. 3b). The C-terminal tails of both Ned8p and ubiquitin possess a large number of essential amino acids. Indeed, seven of the sixteen essential ubiquitin surface residues are located in the C-terminal tail [18], compared to seven of NEDD8's fourteen essential residues. In contrast to ubiquitin, the conserved canonical hydrophobic patch on Ned8p was not strictly essential for *S. pombe* viability, as Leu8 mutants were viable (Fig. 1b) [35]. However, as with ubiquitin, the mutation of Ile44 or Val70 resulted in lethality. As such, both the ubiquitin and Ned8p hydrophobic patches appear to be essential for viability, but the requirement is less strict for Ned8p, as demonstrated by mutation of Leu8. Of the conserved Ned8p residues, which have diverged from ubiquitin, only Leu2, Gly10, Asp18 and Arg25, are essential for viability (Fig. 3a).

## Discussion

NEDD8 shares around 57% identity with ubiquitin, including Lys48, and yet surprisingly not all the essential surface residues are conserved. Polyubiquitination represents a fundamental facet of ubiquitination; and as Ned8p contains eight lysine residues, which are all conserved, this raised the possibility that polyneddylation may represent an important aspect of neddylation. However, although through proteomic analysis we were able to detect NEDD8 chain formation *in vivo*, mutation of all Ned8p lysine residues had no obvious deleterious effect on *S. pombe* growth, either under normal growth conditions or in response to various stress inducing reagents. This suggests that a single moiety is sufficient to fulfil Ned8p function, as in the case for cullin modification [9]. However, we cannot exclude the possibility that Ned8p chains are required, and more prevalent, under specific conditions, not tested here.

The hydrophobic patch of ubiquitin has become synonymous with ubiquitination, mediating the recruitment of numerous proteins via a myriad of interacting domains. The hydrophobic patch of Ned8p is evolutionally conserved amongst all its orthologues. The extended hydrophobic patch Leu8/Ile44/His68/Val70 makes contact with the cullin substrate, along with other N-terminal charged residues [13], [36], and mutation of these residues had been proposed to explain the reduction of *in vitro* ubiquitination of substrates by the neddylated-CRL complex. Our *in vivo* studies have shown that the individual mutation Leu8, his68, and five of these charged residues did not affect viability, with the notable exception of Arg25. Arg25 is known to make direct contact with the NEDD8 activating enzyme [29]. Mutation of Ile44 to alanine, as with the equivalent ubiquitin mutation, resulted in loss of viability, although we did not observe conjugation of the Ned8p mutant protein *in vivo*. As an I44A mutant of NEDD8 was previously shown to conjugate *in vitro* [11], our result is likely due to reduced stability of the mutant protein *in vivo* as no unconjugated form was detected either. Leu2 and Asp18, located on the first and second beta-strand respectively, represent two previously unrecognised residues that are essential of NEDD8 function *in vivo*. Both mutant proteins failed to support viability, despite being able to conjugate *in vivo*, suggesting that other essential facets of neddylation are mediated via these residues. Although we cannot exclude the possibility that these residues are important in mediating deneddylation, it should be noted that *S. pombe* strains deleted for all known deneddylases remain viable. It is further important to note that, *S. pombe* with no detectable levels of cullin-neddylation are still viable, thus, the reduced conjugation efficiency is unlikely to be the cause of the lethality (Girdwood *et al*; manuscript in preparation).

Neddylation has been reported to conjugate to a number of proteins, although the major function of NEDD8 appears to be the modulation of CRL activity via conjugation to cullin proteins. An unresolved question remains how many neddylated substrates exist. The identification of essential residues in Ned8p that do not appear to be involved in this process, suggests that other essential functions remain to be elucidated. Alternatively, these residues may yet have unidentified roles within CRL regulation. Surprisingly, despite the high sequence and functional similarities between ubiquitin and NEDD8, whereas polyubiquitination is essential, the lysine null Ned8p mutant displayed no obvious phenotypes. Although we do not discount the potential of polyneddylation, under certain physiological conditions, it is interesting to note that the only other NEDD8 substrate to be identified in yeast, Lag2p, is as with the cullins, mono-neddylated [37].

## Materials and Methods

### Media and Chemicals

Fission yeast were maintained on rich medium (YES) or on Pombe minimal medium with glutamate (PMG) with appropriate supplements where selection was necessary. General methods for handling the *S. pombe* strains were as described [38]. Nourseothricin (ClonNat) was purchased from Werner Bioagent. 5-Fluoroorotic acid (5-FOA) was purchased from Melford. Strains used in this study are listed in Table 1.

**Table 1:** Strains used in this study.

Strains	Genotype	Derivations
“Wild-type”	*h- ura4 leu1 ade6*	Our stock
*Δned8 diploid*	*h+ ned8::KanMX4 leu1/leu1 ura4/ura4 ade6/ade6*	Bioneer
*Δned8 diploid NAT^r^*	*h+ dcn1::NatMX6 leu1/leu1 ura4/ura4 ade6/ade6*	This study

### Construction of the Ned8 mutants

Sequences for all oligonucleotides used in this study are available as supplementary data (Table S1). Recombination of PCR products via the Gateway™ reaction, were carried out as per manufacturers' instructions (Invitrogen), were initially performed into the pDONR221 vector. Subsequently, pDONR221 inserts were transferred to pDUAL-FFH41c or pDUAL-HFF1c, as per the manufacturers' instructions. Ned8 point mutants were generated via the QuickChange™ site-directed mutagenesis (Stratagene), using either pDUAL-FFH41c-Ned8_GG_ or pDUAL-HFF1c-Ned8_GG_ as template. All mutations were confirmed by automated DNA sequencing.

### Analysis of the ability of mutant Ned8ps to support viability of *S. pombe*

A stable diploid with *ned8^+^* open reading frame replaced by the KanMX4 module was purchased (Bioneer). The KanMX4 module was subsequently replaced by homologous recombination for with the NAT resistance marker (natMX6) [39]. This strain was transformed with pREP42-*ned8* ^+^, and the stable diploid was haplodised by thiabendazole treatment. The resulting strain, with the *ned8^+^* replaced by natMX6 and episomal pREP42- *ned8* ^+^, was transformed with lineralised pDUAL-FFH41c ned8_GG_ and mutant variants thereof. Strainswere grown overnight in PMG-uracil-leucine medium at 25°C. Subsequently, cells were washed in PMG without supplements and spotted onto PMG-uracil-leucine medium, PMG-leucine medium, or PMG-leucine medium supplemented with 2 mg/ml FOA. Plates were left to grow for 5 days at 25°C. Initial spots contained 1×10^5^ cells, subsequent spots stepwise were 10-fold serial dilutions.

### Tissue culture and sample preparation

Logarithmically growing *S. pombe* cultures (10^8^) were harvested, and extracts prepared using trichloroacetic acid (TCA) [40]. Tissue culture transfections were performed with calcium phosphate to transfect H1299 [3] cells in 10 cm dishes, and His_6_-NEDDylated proteins were purified as previously described [32]. Samples were analysed by SDS-polyacrylamide gel electrophoresis (PAGE) (10%, 4–12%, or 3–8% as indicated) followed by Western blot analysis. Ned8p and His_6_-NEDD8 was detected by polyclonal anti-NEDD8 (Alexis, ALX-210-194); the HA, FLAG, and MYC epitopes were detected with monoclonal anti-HA, anti-FLAG and anti-MYC respectively (SIGMA). p53 was detected with the monoclonal DO-1 antibody [3].

## Supporting Information

Table S1 Supplementary materials and methods.
(XLS)
